# Establishment and Application of a Multiple Cross Displacement Amplification Coupled With Nanoparticle-Based Lateral Flow Biosensor Assay for Detection of *Mycoplasma pneumoniae*

**DOI:** 10.3389/fcimb.2019.00325

**Published:** 2019-09-23

**Authors:** Yacui Wang, Yi Wang, Shuting Quan, Weiwei Jiao, Jieqiong Li, Lin Sun, Yonghong Wang, Xue Qi, Xingyun Wang, Adong Shen

**Affiliations:** Key Laboratory of Major Diseases in Children, Ministry of Education, National Key Discipline of Pediatrics (Capital Medical University), National Clinical Research Center for Respiratory Diseases, Beijing Key Laboratory of Pediatric Respiratory Infection Diseases, Beijing Pediatric Research Institute, Beijing Children's Hospital, Capital Medical University, Beijing, China

**Keywords:** *Mycoplasma pneumonia*, multiple cross displacement amplification, nanoparticle-based biosensor, lateral flow biosensor, MCDA–LFB

## Abstract

*Mycoplasma pneumoniae* (*M. pneumoniae*) is responsible for pneumonia, and is a causative agent of other respiratory tract infections (e.g., bronchiolitis and tracheobronchitis). Herein, we established and applied a multiple cross displacement amplification (MCDA) coupled with a nanoparticle-based lateral flow biosensor (LFB) assay (MCDA–LFB) for rapid, simple, and reliable detection of target pathogen. A set of 10 primers was designed based on *M. pneumoniae*-specific P1 gene, and optimal reaction conditions were found to be 30 min at 65°C. The detection results were visually reported using a biosensor within 2 min. The *M. pneumoniae*–MCDA–LFB method specifically detected only *M. pneumoniae* templates, and no cross-reactivity was generated from non-*M. pneumoniae* isolates. The analytical sensitivity for this assay was 50 fg of genomic templates in the pure cultures, as obtained from colorimetric indicator and real-time turbidimeter analysis. The assay was applied to 197 oropharyngeal swab samples collected from children highly suspected of *M. pneumoniae* infection, and compared to culture-based method and real-time PCR assay. The detection rates of *M. pneumoniae* using a culture-based method, real-time PCR assay, and MCDA–LFB assay were 8.1%, 33.0%, and 52.3%, respectively, which indicated that the MCDA–LFB assay was superior to the culture-based method and real-time PCR method for detection of target agent. Using this protocol, 25 min for rapid template extraction followed by MCDA reaction (30 min) combined with LFB detection (2 min) resulted in a total assay time of ~60 min. In conclusion, the MCDA–LFB assay established in this report was a simple, rapid, sensitive, and reliable assay to detect *M. pneumoniae* strains, and can be used as a potential diagnostic tool for *M. pneumoniae* in basic and clinical laboratories.

## Introduction

*Mycoplasma pneumoniae* (*M. pneumoniae*) is one of the most common pathogens causing community-acquired pneumonia (CAP) in children and adolescents (Waites and Talkington, 2004; Atkinson et al., 2008). During epidemics, this causative agent can cause 40% and 70% of CAP in populations with general and high population density, respectively (Loens et al., 2010; Jacobs et al., 2015). Clinical manifestations of *M. pneumoniae* infection are diverse, ranging from mild respiratory symptoms to severe pneumonia, and about 25% of the patients suffered from extrapulmonary complications, including encephalitis, hemolytic anemia, dermatological disorders, etc. (Davis et al., 1988; Taylor-Robinson, 1996; Daxbock et al., 2001; Waites and Talkington, 2004; Defilippi et al., 2008). However, the confirmation of *M. pneumoniae* infection cannot be determined by clinical presentations, which are similar to that of other pathogen infections. Owing to lack of cell walls, *M. pneumoniae* is not sensitive to β-lactam antibiotics, which are selected first for treatment of common pathogens. Thus, developing a simpler, faster, and more accurate method for *M. pneumoniae* detection is imperative to treat timely with effective antibiotics.

Traditionally, there are three techniques for *M. pneumoniae* identification, including culture-based method, serological test, and nucleic acid amplification technology. Culture-based method is laborious, insensitive, and time-consuming, taking 2–4 weeks to generate results, and is not recommended for clinical application (Ieven et al., 1996; Waites et al., 2017). Reliable results of serological test for *M. pneumoniae* detection depend on a 4-fold or greater increase in antibody of paired serums from acute and convalescent phases of the disease with an interval of 2 weeks and also rely on the sample collecting time as well as kits used for serological test, which limit its use in early stages of the disease (Waites et al., 2017). Nucleic acid amplification methods, such as PCR-based assays (e.g., conventional PCR, and real-time PCR), displayed high sensitivity and specificity for *M. pneumoniae* detection. However, PCR-based methods require sophisticated operation, expensive apparatus, and trained personnel, and are not suitable for application in grassroots level (Yuan et al., 2018). Recently, isothermal amplification techniques, such as loop-mediated isothermal amplification (LAMP), have been established and applied for rapid detection of *M. pneumoniae*. However, the interpretation of LAMP result relies on optical instrument analysis (such as real-time turbidity) and colorimetric indicator test, which were costly and subjective, respectively.

To overcome these shortcomings posed by conventional detection technique, we employed a novel isothermal amplification technique, termed multiple cross displacement amplification (MCDA), for simple, rapid and reliable diagnostic of *M. pneumoniae* (Wang et al., 2015, 2016c). The MCDA assay requires 10 primers to achieve the sequence-based amplification at a fixed temperature (60–67°C), which binds to 10 regions of target sequences; thus, the MCDA technique has the advantage of rapidity (~20–30 min), sensitivity (several copies), and specificity. In particular, the nanoparticle-based lateral flow biosensor (LFB) has been devised and used for interpreting the results of MCDA (MCDA–LFB assay), which only requires ~2 min for objective report of MCDA results, and is extremely simple, easy to use, and disposable (Wang et al., 2018).

Here, the first MCDA–LFB assay for the detection of *M. pneumoniae* was developed and verified based on the target sequence of the P1 gene. The analytical sensitivity and specificity in pure culture and clinical samples were determined by comparison with that of culture-based and real-time PCR assays.

## Methods and Materials

### Reagents and Instruments

Isothermal amplification kits, nanoparticle-based lateral flow biosensor, and colorimetric indicator (Te) were purchased from BeiJing-HaiTaiZhengYuan Technology Co., Ltd (Beijing, China). Primers and labeled primers used in this study were synthesized by Tianyi Huiyuan Biotechnology Co., Ltd (Beijing, China). Real-time turbidimeter LA-320C was purchased from Eiken Chemical Co., Ltd, Japan. The PCR instrument was purchased from Beijing Dongsheng Innovation Biotechnology Co., Ltd.

### Bacterial Strains and Clinical Specimens

The *M. pneumoniae* reference strain (M129) and 67 isolated strains (including 46 *M. pneumoniae* strains, 6 other species of *Mycoplasma*, and 15 common agents of respiratory tract) were used for sensitivity and specificity determination (Table 1). Oropharyngeal swab specimens were collected from 197 children in Beijing Children's Hospital from October to November in 2018 with acute respiratory infection highly suspected of *M. pneumoniae* infection, and those who were characterized by clinical presentations (fever, cough, productive sputum, dyspnea, chest pain or abnormal breath sounds); laboratory tests including normal white blood cell count or mild elevation of C-reactive protein; and radiographic findings, such as consolidation, interstitial changes, and pleural effusion. Genomic DNA was extracted from oropharyngeal swab samples using QIAamp DNA Mini Kit according to the manufacturer's instructions. *M. pneumoniae* M129 template DNA was used for primer confirmation, optimal temperature and time verification, and sensitivity and specificity evaluation. The extracted DNA was stored at −20°C before use.

**Table 1:** Bacterial strains used in this study.

**Bacteria**	**Strain no. (source of strains)^**b**^**	**No. of strains**	***M. pneumoniae*–MCDA–LFB^**c**^**
*Mycoplasma pneumoniae*	M129	1	P
	Isolated strains (BCH)	46	P
*Mycoplasma genitalium*^a^	ATCC33530	1	N
*Mycoplasma orale*^a^	ATCC23714	1	N
*Mycoplasma hominis*^a^	ATCC23114	1	N
*Mycoplasma penetrans*^a^	ATCC55252	1	N
*Mycoplasma primatum*^a^	ATCC25960	1	N
*Ureaplasma urealyticum*^a^	ATCC27813	1	N
*Mycobacterium tuberculosis*	Isolated strain (BCH)	1	N
*Klebsiella pneumoniae*	Isolated strain (BCH)	1	N
*Streptococcus pneumoniae*	Isolated strain (BCH)	1	N
*Pseudomonas aeruginosa*	Isolated strain (BCH)	1	N
*Staphylococcus epidermidis*	Isolated strain (BCH)	1	N
*Staphylococcus aureus*	Isolated strain (BCH)	1	N
*Bordetella pertussis*	Isolated strain (BCH)	1	N
*Haemophilus influenzae*	Isolated strain (BCH)	1	N
*Stenotrophomonas maltophilia*	Isolated strain (BCH)	1	N
*Acinetobacter baumannii*	Isolated strain (BCH)	1	N
*Legionella pneumophila*	Isolated strain (BCH)	1	N
*H1N1 influenza*	Isolated strain (BCH)	1	N
*H3N2 influenza*	Isolated strain (BCH)	1	N
*H5N1 influenza*	Isolated strain (BCH)	1	N
*H7N9 influenza*	Isolated strain (BCH)	1	N

a *Bacterial strains were kindly provided by Prof. Fei Zhao, National Institute for Communicable Disease Control and Prevention, Chinese Center for Disease Control and Prevention, Changping, Beijing 102206, PR, China*.

b M129, M. pneumoniae reference strains; BCH, Beijing Children's Hospital; CDC, Chinese Center for Disease Control and Prevention; ATCC, American Type Culture Collection.

c *P, positive; N, negative. Only M. pneumoniae could be detected by M. pneumoniae–MCDA–LFB assay, indicating the extremely high selectivity of the method*.

### Primer Design

**Figure 1:**
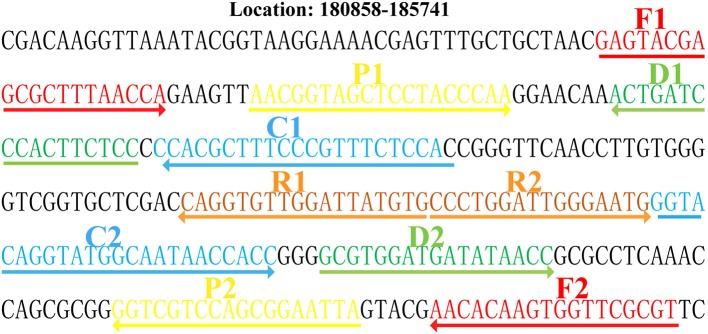
Locations and sequences of the primers on the P1 gene. Location and sequence of the P1 gene (*M. pneumoniae*-specific gene) used to design multiple cross displacement amplification (MCDA) primers. The locations of the P1 gene of *M. pneumoniae* was from 180,858 to 185,741. The nucleotide sequence of the sense strand of the P1 gene is shown. Right arrows and left arrows indicate sense and complementary sequences that were used.

A set of 10 primers spanning 10 different regions of the target gene fragment, including two displacement (F1 and F2) primers, two cross (CP1 and CP2) primers, and six amplification (C1^*^, C2, D1^*^, D2, R1, and R2) primers, was designed based on the specific P1 gene of *M. pneumoniae* using Primer Premier 5.0. More details of the primer design and working principles of the MCDA assay have been published in the previous publication (Wang et al., 2015). The specific primers obtained were subjected to sequence alignment analysis in NCBI database to exclude non-specific matching with other pathogens and then the optimized primers were acquired. The primer sequences, locations, and modifications are shown in Figure 1 and Table 2.

**Table 2:** Primers used in this study.

**Primers^**a**^**	**Sequences and modifications**	**Gene positions^**b**^**	**Length^**c**^**
F1	5′-GAGTACGAGCGCTTTAACCA-3′	183,141–183,160	20 nt
F2	5′-ACGCGAACCACTTGTGTT-3′	183,384–183,401	18 nt
CP1	5′-TGGAGAAACGGGAAAGCGTGGAACGGTAGCTCCTACCCAA-3′		40 mer
CP2	5′-GGTACAGGTATGGCAATAACCACCTAATTCCGCTGGACGACC-3′		42 mer
C1*	5'-FITC-TGGAGAAACGGGAAAGCGTGG-3′	183,211–183,231	21 nt
C2	5′-GGTACAGGTATGGCAATAACCACC-3′	183,298–183,321	24 nt
D1*	5′-Biotin-GGAGAAGTGGGATCAGT-3′	183,193–183,209	17 nt
D2	5′-GCGTGGATGATATAACC-3′	183,325–183,341	17 nt
R1	5′-CACATAATCCAACACCTG-3′	183,264–183,281	18 nt
R2	5′-CCCTGGATTGGGAATG-3′	183,282–183,297	16 nt
P1	5′-AACGGTAGCTCCTACCCAA-3′	183,167–183,185	19 nt
P2	5′-TAATTCCGCTGGACGACC-3′	183,361–183,378	18 nt

a FITC, fluorescein isothiocyanate. C1^*^, 5′-labeled with FITC when used in the MCDA–LFB assay; D1^*^, 5′-labeled with biotin when used in the MCDA–LFB assay.

b *The primer position is based on the sequence M. pneumoniae M129 with NCBI Reference Sequence: NC-000912.1*.

c *nt, nucleotide; mer, monomeric*.

### The Standard MCDA–LFB Assay

The MCDA–LFB assay was conducted on a volume of 25 μl of reaction mixtures, containing 0.4 μM each of displacement primers (F1 and F2), 0.8 μM each of amplification primers (C1^*^, C2, R1, R2, D1^*^, and D2), 1.6 μM each of cross primers (CP1 and CP2), 12.5 μl of 2 × reaction mix (HaiTaiZhengYuan, Beijing, China), 1 μl of 10 U Bst DNA polymerase (HaiTaiZhengYuan, Beijing, China), and 1 μl of DNA template. In addition, 1 μl of extracted DNA from non-*M. pneumoniae* was selected as negative controls, and 1 μl of double distilled water was used as blank controls. The amplification reactions were incubated at 65°C for 1 h, and colorimetric reagents and LFB were used for amplification product detection. A volume of 0.5 μl of MCDA product was used for reporting the result by LFB.

### The Optimal Reaction Temperature of the MCDA–LFB Assay

To determine the optimal reaction temperature of the MCDA–LFB assay during the amplification stage, amplification mixtures with 1 μl DNA of M129 (500 pg/μl) were incubated at a constant temperature ranging from 61 to 68°C at 1°C increments monitored by a real-time turbidimeter for 40 min. Mixtures with 1 μl of non-*M. pneumoniae* genomic DNA and 1 μl of double distilled water were used as negative controls and blank controls, respectively.

### The Analytical Sensitivity and Specificity of the MCDA–LFB Assay in Pure Culture

For specificity evaluation of the MCDA–LFB assay, genomic templates were extracted from 46 *M. pneumoniae* strains and 21 non-*M. pneumoniae* strains [*Mycoplasma genitalium* (ATCC33530), *Mycoplasma orale* (ATCC23714), *Mycoplasma hominis* (ATCC23114), *Mycoplasma penetrans* (ATCC55252), *Mycoplasma primatum* (ATCC25960), *Ureaplasma urealyticum* (ATCC27813), *Mycobacterium tuberculosis, Klebsiella pneumoniae, Streptococcus pneumoniae, Pseudomonas aeruginosa, Staphylococcus epidermidis, Staphylococcus aureus, Bordetella pertussis, Haemophilus influenzae, Stenotrophomonas maltophilia, Acinetobacter baumannii, Legionella pneumophila, H1N1 influenza, H3N2 influenza, H5N1 influenza*, and *H7N9 influenza*]. Each sample was tested at least twice. Serial dilutions (5 ng, 500 pg, 50 pg, 5 pg, 500 fg, 50 fg, and 5 fg) of *M. pneumoniae* M129 genomic DNA in pure culture were prepared to determine the limit of detection (LoD) of the MCDA–LFB assay. The amplifications of MCDA were monitored by a colorimetric indicator and LFB, and three replicates of each dilution were conducted to determine the analytical sensitivity. In order to compare the analytical sensitivity of the MCDA–LFB assay with that of the real-time PCR method, the same dilutions of *M. pneumoniae* M129 DNA templates were tested by real-time PCR assay.

### The Optimal Amplification Time of MCDA–LFB Assay

The serially diluted templates were applied for optimizing the amplification time during the reaction stage. MCDA reaction mixtures were incubated at the optimal amplification temperature with the reaction time ranging from 10 to 40 min with 10-min intervals. The MCDA results were reported using LFB, and each reaction time was tested at least two replicates.

### Application of MCDA–LFB Assay in Clinical Specimens

To demonstrate the availability of MCDA–LFB assay in clinical specimens, oropharyngeal swab samples collected from 197 children with acute respiratory infection were tested by the MCDA–LFB assay. The results of the MCDA–LFB assay for *M. pneumoniae* detection were compared with that of the culture-based method and real-time PCR assay for the identical samples. *M. pneumoniae* and macrolide-resistant isolate diagnostic kits used for real-time PCR conduction were purchased from Mole Bioscience Co., Ltd (Jiangsu, China).

### Statistical Analysis

Comparison between the three methods of culture, real-time PCR, and MCDA was analyzed by χ^2^ test or Fisher's exact test. SPSS software (version 23) was used for statistical analysis, and *P* < 0.05 was considered statistically significant.

## Results

### Confirmation and Detection of *M. pneumoniae*–MCDA–LFB Amplifications

**Figure 2:**
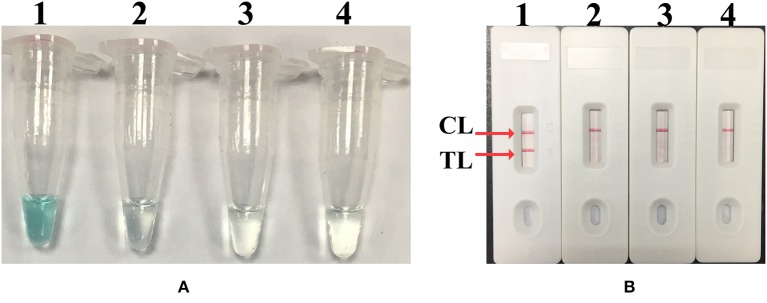
Confirmation and identification of MCDA products. **(A)** The detection of MCDA products by colorimetric indicators: tube 1, positive amplification of *M. pneumoniae* strain; tube 2, negative control of *Streptococcus pneumoniae* strain; tube 3, negative control of *Staphylococcus aureus* strain; tube 4, blank control of double distilled water. **(B)** Lateral flow biosensor used for MCDA products: biosensor 1, positive amplification (*M. pneumoniae*); biosensor 2, negative control (*S. pneumoniae*); biosensor 3, negative control (*S. aureus*); biosensor 4, blank control (double distilled water). TL, test line; CL, control line.

The MCDA reaction was performed in the presence and absence of *M. pneumoniae* genomic DNA at a constant temperature (65°C) for 40 min to determine the feasibility of the primers designed. An LFB and a colorimetric indicator were used to confirm *M. pneumoniae*–MCDAs. Using a colorimetric indicator, the positive amplification changed into light green, while the colorlessness was seen in the negative and blank controls (Figure 2A). Using an LFB, two visible red lines (Test line, TL; Control line, CL) were seen in MCDA-positive products; only one red line (CL) was observed in negative and blank controls (Figure 2B). These results suggested that the MCDA primer set designed here was a good candidate for development of MCDA-based assay for *M. pneumoniae* detection.

### Optimal Temperature of *M. pneumoniae*–MCDA–LFB Assay

**Figure 3:**
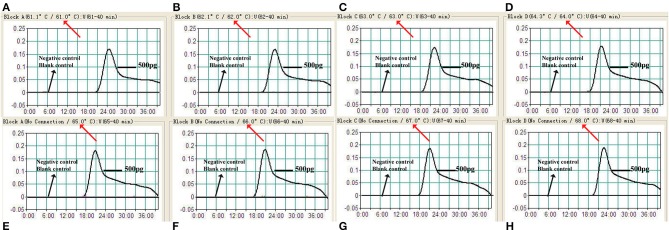
Temperature optimization for MCDA assay. Real-time turbidimeter was applied for MCDA reactions, and the corresponding curves of concentrations of *M. pneumoniae* (M129) genomic DNA were demonstrated in the picture. A turbidity of >0.1 indicated positive amplification of MCDA. Eight kinetic graphs **(A–H)** were obtained at distinct temperatures ranging from 61 to 68°C with DNA templates of *M. pneumoniae* at a level of 500 pg/μl per reaction.

In order to optimize the temperature of the MCDA–LFB assay for *M. pneumoniae* detection, MCDA reactions were performed at eight different temperatures ranging from 61 to 68°C at 1°C intervals for 40 min. As shown in Figure 3, 65°C was the optimal temperature for *M. pneumoniae*–MCDA–LFB amplification, because a threshold value of 0.1 of absorbance that indicated positive amplification from the *M. pneumoniae*–MCDA reaction was reached most quickly at 65°C. Herein, the optimal temperature of 65°C was employed for the subsequent *M. pneumoniae*–MCDA–LFB examinations conducted in this report.

### Sensitivity of the *M. pneumoniae*–MCDA–LFB Assay

**Figure 4:**
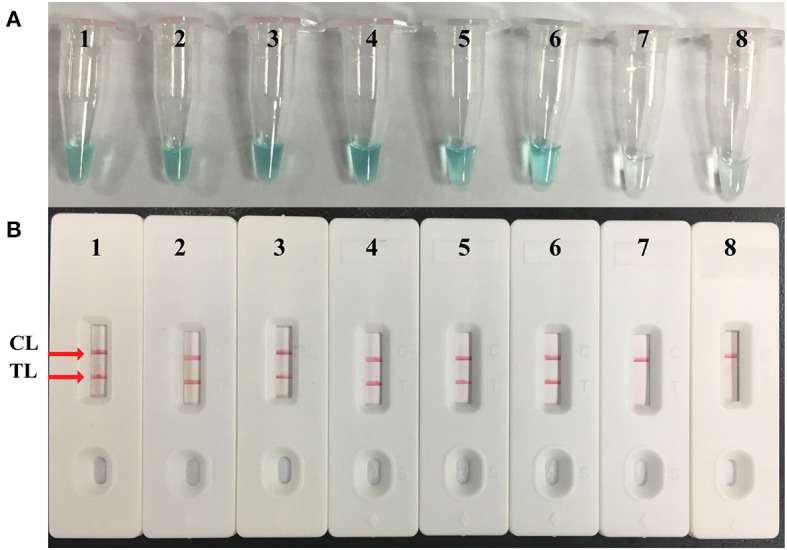
Sensitivity confirmation of *M. pneumoniae*–MCDA assay. Two monitoring assays including **(A)** colorimetric indicator and **(B)** lateral flow biosensor were applied for MCDA products. Serial dilutions of *M. pneumoniae* (M129) genomic DNA 5 ng, 500 pg, 50 pg, 5 pg, 500 fg, 50 fg, and 5 fg were used for sensitivity analysis. Tubes/strips 1–7 represented different concentrations of DNA, 5 ng, 500 pg, 50 pg, 5 pg, 500 fg, 50 fg, and 5 fg per reaction; tube/strip 8 represented blank control. TL, test line; CL, control line.

Serial dilutions of *M. pneumoniae* M129 genomic DNA (5 ng, 500 pg, 50 pg, 5 pg, 500 fg, 50 fg, and 5 fg) were used to confirm the LoD of the *M. pneumoniae*–MCDA–LFB assay. By LFB, the LoD of *M. pneumoniae*–MCDA was 50 fg per reaction (Figure 4B). In parallel, the analytical sensitivity was 50 fg per reaction using colorimetric indicator, which was completely consistent with LFB analysis. Compared with real-time PCR for analytical sensitivity, the MCDA–LFB assay was 10-fold more sensitive than real-time PCR technique for *M. pneumoniae* detection (Figure S1).

### Specificity Analysis of the *M. pneumoniae*–MCDA–LFB Assay

Genomic DNA extracted from 46 *M. pneumoniae* isolates and 21 non-*M. pneumoniae* strains were used for MCDA–LFB detection under the optimal conditions confirmed above. As monitored by LFB, the positive products were seen in *M. pneumoniae* strains with two red lines (TL and CL) observed on the biosensor (Figure S2). There was no cross-reactivity in non-*M. pneumoniae* strains, and only one red line (CL) appeared on the LFB (Figure S2). These results demonstrated that the analytical specificity of the *M. pneumoniae*–MCDA–LFB assay for *M. pneumoniae* detection was 100%.

### Optimal Time of the *M. pneumoniae*–MCDA–LFB Assay

**Figure 5:**
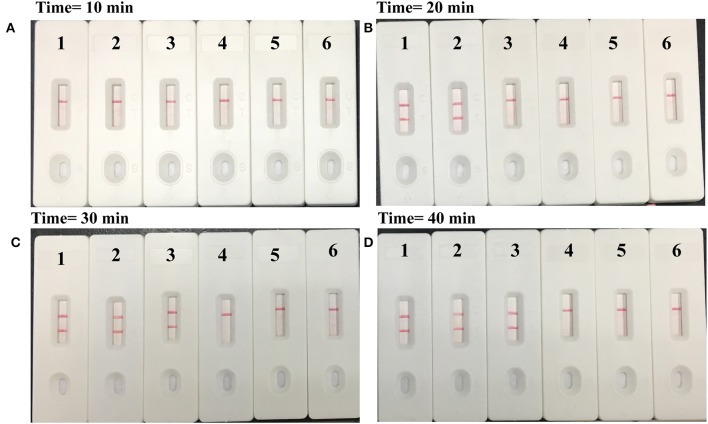
Time optimization for *M. pneumoniae*-MCDA assay. The standard MCDA reaction with four distinct times including **(A)** 10 min, **(B)** 20 min, **(C)** 30 min, and **(D)** 40 min were tested and analyzed by lateral flow biosensors at the optimal temperature of 65°C. Strips 1–4 represented genomic DNA at the level of 5 pg, 500 fg, 50 fg, and 5 fg, respectively; strip 5, negative control (*S. pneumoniae*); strip 6, blank control (double distilled water).

In order to assess the optimal amplification time for *M. pneumoniae* detection, reaction times were increased from 10 to 40 min at 10-min intervals at 65°C. The results indicated that 30 min was sufficient for the MCDA–LFB assay, because the genomic template at the LoD level could be detected. Two red lines including TL and CL appeared on the LFB (Figure 5).

### Application of the MCDA–LFB Assay in Clinical Specimens for *M. pneumoniae* Detection

In order to further verify whether the MCDA–LFB assay could be implemented in clinical samples, 197 oropharyngeal swab specimens were divided into three equal parts, and then were simultaneously detected by the culture-based method, MCDA–LFB assay, and real-time PCR. Of the 197 oropharyngeal swab samples, 103 (52.3%) samples were detected positive by the MCDA–LFB assay, and only 16 (8.1%) and 55 (33.0%) cases were confirmed positive by the culture-based method and real-time PCR test, which indicated that the MCDA–LFB assay was more sensitive than the culture-based method and real-time PCR assay (*P* = 0.000) (Table 3).

**Table 3:** Comparison of culture-based method, MCDA–LFB assay, and real-time PCR for *M. pneumoniae* detection.

**Detection assays**	**Oropharyngeal swab specimens (*****N*** **=** **197)**		***P***
	**Positive**	**Negative**	
Culture	16 (8.1)	181 (91.9)	0.000
Real-time PCR	65 (33.0)	132 (67.0)	
MCDA–LFB	103 (52.3)	94 (47.7)	

*Data were presented as number (%)*.

*P-values < 0.05 were considered statistically significant*.

## Discussion

Up to now, MCDA combined with lateral flow biosensor methodology (MCDA–LFB) had been successfully applied for rapid detection of various pathogens, such as *Vibrio parahaemolyticus, Leptospira interrogans, Shigella* spp., *K. pneumoniae*, etc. (Wang et al., 2016a,b; Niu et al., 2018; Li et al., 2019), and these reports demonstrated that the MCDA–LFB assay was a valuable and powerful tool for simple, rapid, and reliable detection of target sequence. In this study, we established a new MCDA–LFB assay for *M. pneumoniae* detection and verified its availability using pure cultures and clinical samples. The merits of the MCDA assay are rapidity, simplicity, sensitivity, and specificity, and the amplification products can be detected from as little as three bacterial cells (Wang et al., 2016a). Moreover, our assay can be conducted just by a heater or water bath that were easily acquired and more portable, avoiding the use of sophisticated apparatus compared with PCR-based techniques. In particular, only a shorter isothermal time (30 min) was required for the MCDA–LFB assay during the reaction stage; thus, the whole process, including rapid template preparation (25 min), isothermal amplification (30 min), and reporting of results (within 2 min), can be finished within 60 min (Figure 5). Hence, the *M. pneumoniae*–MCDA–LFB assay developed here has great advantages for point-of-care testing in clinical settings.

A set of 10 *M. pneumoniae*–MCDA primers were designed targeting the specific P1 gene, which ensures high selectivity for sequence detection. Then, the analytical specificity of the MCDA–LFB assay for *M. pneumoniae* detection was successfully confirmed with genomic DNA extracted from 46 *M. pneumoniae* isolates and 21 non-*M. pneumoniae* strains in pure culture. The positive amplifications were displayed in *M. pneumoniae* isolates but not in non-*M. pneumoniae* strains (Figure S2), which indicated that the MCDA–LFB assay was reliable for target pathogen detection.

Apart from high specificity of the MCDA–LFB assay, the excellent sensitivity of the MCDA–LFB assay was also demonstrated. As shown in Figure 4, MCDA–LFB was capable of detecting as little as 50 fg of genomic DNA in pure culture per reaction, which was in accordance with the colorimetric indicator confirmed in our study. In this report, the *M. pneumoniae*–MCDA–LFB assay proved to be more sensitive than the culture-based method and real-time PCR in clinical specimens (Table 3). The possible reasons for the lower detection rate of *M. pneumoniae* using the culture-based method are as follows: high nutritional requirements for growing, strict requirements for laboratories and trained personnel, greater vulnerability to the environment due to the lack of cell walls, and most importantly the application of drugs leading to the death of pathogens. The lower sensitivity of the real-time PCR method depended on the LoD (500 fg) of the kits used in this report, as described in Figure S1. Also, previous studies demonstrated that the isothermal amplification method was less affected by inhibitors in the samples (Wang et al., 2014; Petrone et al., 2015). Furthermore, the cost of this assay is affordable: US$3.5 and US$2 for MCDA and LFB, respectively (Wang et al., 2016a). Thus, MCDA–LFB assays are more suitable for clinical application for its simplicity, rapidity, and low cost.

In particular, a biosensor was used for the reporting of MCDA results. To identify the detectable MCDA amplicons by LFB, C1 and D1 primers were labeled with FITC and biotin at the 5′ end, respectively. The double-labeled MCDA products, which were FITC- and biotin-labeled, were formed during the reaction amplification stage. One end of the positive amplicons labeled with FITC was captured by anti-FITC antibody on the TL of the LFB, and the other end labeled with biotin was combined with nanoparticles for visualization. The excessive nanoparticles were captured by biotinylated bovine serum albumin on the CL of the LFB, indicating the availability of the LFB. Thus, the positive *M. pneumoniae*–MCDA amplicons were successfully detected by the LFB and displayed two red lines (TL and CL) on the biosensor within 2 min, while only one red line (CL) was displayed in negative controls and blank controls (Figures 2, 4, 5). There were other monitoring techniques for the MCDA interpretation, such as agarose gel electrophoresis, real-time turbidimeter, and colorimetric indicator. The product of MCDA is a complex mixture of different sizes of amplicons; thus, agarose gel electrophoresis and colorimetric indicator couldn't distinguish the specific and non-specific amplicons (Ge et al., 2013). Reading of MCDAs by agarose gel electrophoresis depends on sophisticated instruments and trained personnel, limiting its application in poor-resource areas. Colorimetric indicator was somewhat unreliable due to the insufficient amplification products, resulting in ambiguous judgment. By using a real-time turbidimeter, it could be easily affected by background interference. Therefore, the LFB assay is the preferred candidate for interpretation of the MCDA results, considering the advantages of the LFB assay illustrated above.

## Conclusion

In this report, we successfully developed a reliable MCDA–LFB assay for *M. pneumoniae* detection, which could detect *M. pneumoniae* in time and assist clinicians making the right decisions. The MCDA–LFB assay established here is rapid, simple, sensitive, specific, and cost-effective compared with culture-based and PCR-based methods. Therefore, the MCDA–LFB assay with advantages mentioned above is more suitable for a wide application in various medical institutions, especially in resource-limited areas.

## Data Availability Statement

The raw data supporting the conclusions of this manuscript will be made available by the authors, without undue reservation, to any qualified researcher.

## Ethics Statement

Our study was approved by the Ethical Committee of Beijing Children's Hospital, and guardians of the enrolled children signed informed consent documents

## Author Contributions

YW, YCW, and AS conceived and designed the experiments. YW, YCW, AS, JL, WJ, LS, YHW, XQ, XW, and SQ performed the experiments. YW, YCW, AS, JL, WJ, LS, YHW, XQ, XW, and SQ contributed the reagents and materials. YCW and AS analyzed the data. YCW and YW performed the software. YW, YCW, and AS wrote the paper.

### Conflict of Interest

The authors declare that the research was conducted in the absence of any commercial or financial relationships that could be construed as a potential conflict of interest.

## References

[B1] Atkinson T. P. Balish M. F. Waites K. B. (2008). Epidemiology, clinical manifestations, pathogenesis and laboratory detection of *Mycoplasma pneumoniae* infections. FEMS Microbiol. Rev. 32, 956–973. 10.1111/j.1574-6976.2008.00129.x 18754792

[B2] Davis C. P. Cochran S. Lisse J. Buck G. DiNuzzo A. R. Weber T.. (1988). Isolation of *Mycoplasma pneumoniae* from synovial fluid samples in a patient with pneumonia and polyarthritis. Arch. Intern. Med. 148, 969–970. 10.1001/archinte.1988.00380040209029 3128197

[B3] Daxbock F. Zedtwitz-Liebenstein K. Burgmann H. Graninger W. (2001). Severe hemolytic anemia and excessive leukocytosis masking *Mycoplasma pneumoniae*. Ann. Hematol. 80, 180–182. 10.1007/s002770000250 11320906

[B4] Defilippi A. Silvestri M. Tacchella A. Giacchino R. Melioli G. Di Marco E.. (2008). Epidemiology and clinical features of *Mycoplasma pneumoniae* infection in children. Respir. Med. 102, 1762–1768. 10.1016/j.rmed.2008.06.022 18703327

[B5] Ge Y. Wu B. Qi X. Zhao K. Guo X. Zhu Y.. (2013). Rapid and sensitive detection of novel avian-origin *influenza A* (*H7N9*) virus by reverse transcription loop-mediated isothermal amplification combined with a lateral-flow device. PLoS ONE 8:e69941. 10.1371/journal.pone.0069941 23936359 PMC3731295

[B6] Ieven M. Ursi D. Van Bever H. Quint W. Niesters H. G. Goossens H. (1996). Detection of *Mycoplasma pneumoniae* by two polymerase chain reactions and role of *M. pneumoniae* in acute respiratory tract infections in pediatric patients. J. Infect. Dis. 173, 1445–1452. 10.1093/infdis/173.6.1145 8648218

[B7] Jacobs E. Ehrhardt I. Dumke R. (2015). New insights in the outbreak pattern of *Mycoplasma pneumoniae*. Int. J. Med. Microbiol. 305, 705–708. 10.1016/j.ijmm.2015.08.021 26319941

[B8] Li S. Liu Y. Chen X. Wang M. Hu W. Yan J. (2019). Visual and rapid detection of *Leptospira interrogans* using multiple cross-displacement amplification coupled with nanoparticle-based lateral flow biosensor. Vector Borne Zoonotic Dis. 19, 604–612. 10.1089/vbz.2018.2395 30702382

[B9] Loens K. Goossens H. Ieven M. (2010). Acute respiratory infection due to *Mycoplasma pneumoniae*: current status of diagnostic methods. Eur. J. Clin. Microbiol. Infect. Dis. 29, 1055–1069. 10.1007/s10096-010-0975-2 20526788 PMC7088226

[B10] Niu L. Zhao F. Chen J. Nong J. Wang C. Gao N.. (2018). Isothermal amplification and rapid detection of *Klebsiella pneumoniae* based on the multiple cross displacement amplification (MCDA) and gold nanoparticle lateral flow biosensor (LFB). PLoS ONE 13:e0204332. 10.1371/journal.pone.0204332 30273362 PMC6166938

[B11] Petrone B. L. Wolff B. J. DeLaney A. A. Diaz M. H. Winchell J. M. (2015). Isothermal detection of *Mycoplasma pneumoniae* directly from respiratory clinical specimens. J. Clin. Microbiol. 53, 2970–2976. 10.1128/JCM.01431-15 26179304 PMC4540912

[B12] Taylor-Robinson D. (1996). Infections due to species of *Mycoplasma* and *Ureaplasma*: an update. Clin. Infect Dis. 23, 671–682. 10.1093/clinids/23.4.671 8909826

[B13] Waites K. B. Talkington D. F. (2004). *Mycoplasma pneumoniae* and its role as a human pathogen. Clin. Microbiol. Rev. 17, 697–728. 10.1128/CMR.17.4.697-728.2004 15489344 PMC523564

[B14] Waites K. B. Xiao L. Liu Y. Balish M. F. Atkinson T. P. (2017). *Mycoplasma pneumoniae* from the respiratory tract and beyond. Clin. Microbiol. Rev. 30, 747–809. 10.1128/CMR.00114-16 28539503 PMC5475226

[B15] Wang Y. Li H. Li D. Li K. Wang Y. Xu J.. (2016a). Multiple cross displacement amplification combined with gold nanoparticle-based lateral flow biosensor for detection of *Vibrio parahaemolyticus*. Front. Microbiol. 7:2047. 10.3389/fmicb.2016.02047 28066368 PMC5177632

[B16] Wang Y. Li H. Wang Y. Xu H. Xu J. Ye C. (2018). Antarctic thermolabile uracil-DNA-glycosylase-supplemented multiple cross displacement amplification using a label-based nanoparticle lateral flow biosensor for the simultaneous detection of nucleic acid sequences and elimination of carryover contamination. Nano Res. 11, 2632–2647. 10.1007/s12274-017-1893-z

[B17] Wang Y. Wang Y. Ma A. Li D. Ye C. (2014). Rapid and sensitive detection of *Listeria monocytogenes* by cross-priming amplification of Imo0733 gene. FEMS Microbiol. Lett. 361, 43–51. 10.1111/1574-6968.12610 25273275

[B18] Wang Y. Wang Y. Ma A. J. Li D. X. Luo L. J. Liu D. X.. (2015). Rapid and sensitive isothermal detection of nucleic-acid sequence by multiple cross displacement amplification. Sci. Rep. 5:11902. 10.1038/srep11902 26154567 PMC4648395

[B19] Wang Y. Wang Y. Xu J. Ye C. (2016b). Development of multiple cross displacement amplification label-based gold nanoparticles lateral flow biosensor for detection of *Shigella* spp. Front. Microbiol. 7:1834. 10.3389/fmicb.01834 27917160 PMC5114309

[B20] Wang Y. Wang Y. Zhang L. Liu D. Luo L. Li H.. (2016c). Multiplex, rapid, and sensitive isothermal detection of nucleic-acid sequence by endonuclease restriction-mediated real-time multiple cross displacement amplification. Front. Microbiol. 7:753. 10.3389/fmicb.2016.00753 27242766 PMC4870240

[B21] Yuan X. Bai C. Cui Q. Zhang H. Yuan J. Niu K.. (2018). Rapid detection of *Mycoplasma pneumoniae* by loop-mediated isothermal amplification assay. Medicine (Baltimore) 97:10806. 10.1097/MD.0000000000010806 29923972 PMC6023700

