# Supplementary figures and images for: Establishment and Application of a Multiple Cross Displacement Amplification Coupled With Nanoparticle-Based Lateral Flow Biosensor Assay for Detection of Mycoplasma pneumoniae
Source: Front Cell Infect Microbiol. 2019 Sep 23;9:325. doi: 10.3389/fcimb.2019.00325 (PMC6767991; doi:10.3389/fcimb.2019.00325)

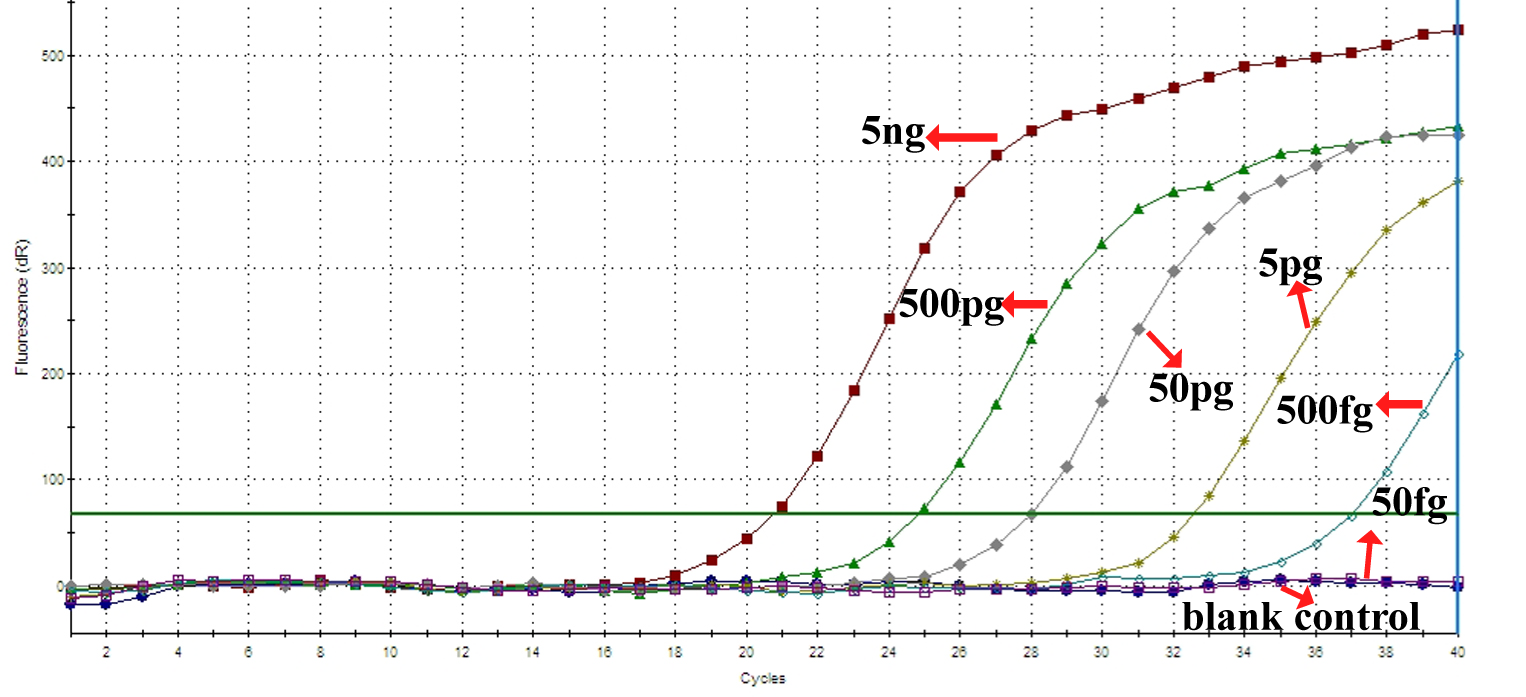

Supplement: Supplementary file 1 [file Image_1.TIF]

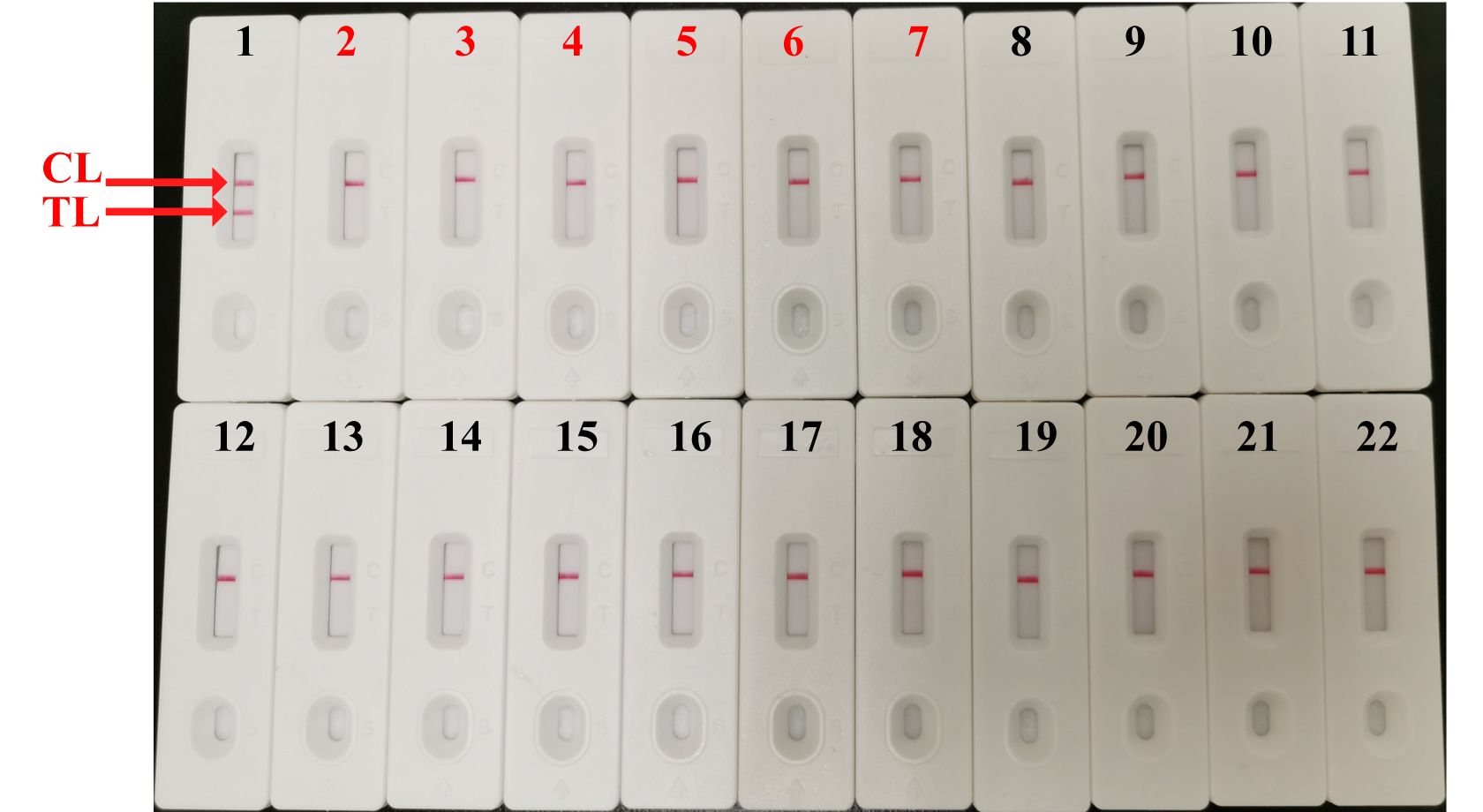

Supplement: Supplementary file 2 [file Image_2.TIF]
