# Supplementary material for: Establishment and Application of a Multiple Cross Displacement Amplification Coupled With Nanoparticle-Based Lateral Flow Biosensor Assay for Detection of Mycoplasma pneumoniae
Source: Front Cell Infect Microbiol. 2019 Sep 23;9:325. doi: 10.3389/fcimb.2019.00325 (PMC6767991; doi:10.3389/fcimb.2019.00325)
Supplement: Supplementary file 3 [file Table_1.doc]

**Supporting information**

**Title page:**

**Establishment and application a** **multiple cross displacement amplification coupled with nanoparticle-based lateral flow biosensor assay for detection of *Mycoplasma pneumoniae***

Yacui Wang, Yi Wang, Shuting Quan, Weiwei Jiao, Jieqiong Li, Lin Sun, Yonghong Wang, Xue Qi, Xingyun Wang, Adong Shen*

^1^ Key Laboratory of Major Diseases in Children, Ministry of Education, National Key Discipline of Pediatrics (Capital Medical University), National Clinical Research Center for Respiratory Diseases, Beijing Key Laboratory of Pediatric Respiratory Infection Diseases, Beijing Pediatric Research Institute, Beijing Children’s Hospital, Capital Medical University, Beijing 10045, China.

Text page:

Figure:

Tables:

Running Title: MCDA-LFB for detection of *M. pneumoniae*

Funding：This work was supported by the Science and technology project of Beijing for glucocorticoid therapy study of pediatric severe mycoplasma pneumoniae pneumonia (Z171100001017081)

Corresponding author: Prof. Adong Shen; Email: shenad18@126.com ; Tel: +86 10 59616898; Fax: +86 10 59718662

**Supplementary Figure legends**

**Figure S1. Analytical sensitivity of real-time PCR for *M. pneumoniae* detection**

Serial dilutions of *M. pneumoniae* (M129) genomic DNA 5 ng, 500 pg, 50 pg, 5 pg, 500 fg, 50 fg, 5 fg were used for sensitivity analysis by real-time PCR.

**Figure S2. Specificity analysis of *M. pneumoniae*-MCDA assay with different strains**

MCDA reactions conducted with distinct DNA templates from 1 *M. pneumoniae* reference strain (M129), 21 non-*M. pneumoniae* agents, and were detected by Lateral flow biosensors. Biosensor 1, *M. pneumoniae* reference strain (M129); biosensors 2-7, *Mycoplasma genitalium* (ATCC33530), *Mycoplasma orale* (ATCC23714), *Mycoplasma hominis* (ATCC23114), *Mycoplasma penetrans* (ATCC55252), *Mycoplasma primatum* (ATCC25960), *Ureaplasma urealyticum* (ATCC27813), biosensors 8-22, *Mycobacterium tuberculosis*, *Klebsiella pneumoniae*, *Streptococcus pneumoniae, Pseudomonas aeruginosa, Staphylococcus epidermidis, Staphylococcus aureus,* *Bordetella pertussis*, *Haemophilus influenzae*, *Stenotrophomonas maltophilia*, *Acinetobacter baumannii*, *Legionella pneumophila*, *H1N1 influenza*, *H3N2 influenza*, *H5N1 influenza*, *H7N9 influenza*
